# *WDR33* alternative polyadenylation is dependent on stochastic poly(a) site usage and splicing efficiencies

**DOI:** 10.1080/15476286.2024.2408708

**Published:** 2024-09-26

**Authors:** Lizhi Liu, Takahiro Seimiya, James L. Manley

**Affiliations:** Department of Biological Sciences, Columbia University, New York, NY, USA

**Keywords:** WDR33, CFIm25, alternative polyadenylation, innate immune responses, polyadenylation site, alternative splicing, CRISPR/Cas13, minigene

## Abstract

Transcripts from the human *WDR33* gene, which encodes a central component of the mRNA polyadenylation (PA) machinery, are subject to alternative polyadenylation (APA) within promoter-proximal introns/exons. This APA, which itself involves usage of multiple PA sites, results in the production of two non-canonical protein isoforms, V2 and V3, that are functionally completely unrelated to the full-length protein, with roles in innate immunity. The mechanism and regulation of *WDR33* APA are unclear. Here, we report that levels of the PA factor CFIm25 modulate V2 and V3 expression, and that PA site usage of both V2 and V3 varies in distinct immune responses. Using newly developed assays to measure splicing and PA site strength, we show that splicing of V2-associated intron 6 is inefficient, allowing V2 to be produced using weak PA sites. Usage of V3’s strong PA sites, on the other hand, is relatively low, reflecting the high efficiency of intron 7 splicing coupled with dependency on usage of an alternative 3’ splice site within the intron. Overall, our findings demonstrate that usage of *WDR33* alternative PA sites is stochastic, dependent on a complex interplay between splicing and PA, and thus provide new insights into mechanisms underlying APA.

## Introduction

Cleavage and polyadenylation (CPA), which refer to the endonucleolytic cleavage of the nascent mRNA at its 3' end and the synthesis of a poly(A) tail, are two coupled reactions required for mRNA biogenesis. In mammals, CPA involves a large number of protein factors that are grouped into four multisubunit complexes, cleavage and polyadenylation specificity factor (CPSF), cleavage stimulation factor (CstF), and cleavage factors (CF) I and II, plus a number of auxiliary factors, including poly(A) polymerase [1]; reviewed by [2]. The region where CPA occurs is known as the poly(A) site (PAS), which is a collection of multiple RNA elements. A hexameric sequence element, consensus A[A/U]UAAA, is a conserved sequence that is typically required for CPA (for reviews see [3] and [4]). This element, often referred to as the ‘hexamer’, is recognized directly by CPSF30 and WDR33, two CPSF subunits [5–8]. Sequences upstream (UGUA) and downstream (G/U-rich) of the hexamer are bound by CFI and CstF, respectively [2]. Just as with transcriptional promoters, PASs thus consist of multiple sequence elements that can vary in composition and thus strength, and function to facilitate assembly of multisubunit protein complexes.

Approximately 70% of mammalian genes harbour multiple PASs [9,10]. Usage of different PASs can produce mRNA isoforms with different coding sequences and/or different 3’ untranslated region (3'UTR) lengths. This phenomenon, known as alternative polyadenylation (APA), is thus a widespread gene expression regulatory mechanism (for reviews, see [2] and [11]. Many CPA factors have been shown to regulate APA. Knockdown of CFIm25 causes 3’UTR shortening and facilitates induced pluripotent stem cell reprogramming [12], whereas downregulation of FIP1, a CPSF subunit, occurs during embryonic stem cell differentiation and causes 3'UTR lengthening [13]. During B cell differentiation, CstF64 is upregulated to promote usage of an intronic PAS of the IgM H-chain gene, producing the secreted form of IgM instead of the membrane-bound form [14,15]. These examples highlight the importance of APA in many biological processes, such as cell differentiation and immune responses.

Human *WDR33* pre-mRNAs are subject to APA within introns/exons, producing three distinct protein isoforms. We recently demonstrated that the two non-canonical isoforms, termed V2 and V3, are functionally completely unrelated to the canonical protein, V1, which is one of the hexamer-recognizing CPSF subunits [16]. V2 harbours a C-terminal transmembrane domain encoded within intron 6, which is required for its localization to the endoplasmic reticulum. There, V2 interacts with STING, an immune protein that induces a variety of immune responses in the presence of cytosolic double-stranded (ds) DNA (for reviews see [17,18]). V2 suppresses STING disulphide bond-mediated oligomerization but facilitates STING-induced autophagy. V3 also interacts with STING, and appears to increase STING protein levels. Despite our knowledge of the functional significance of V2 and V3, the mechanisms and regulation of *WDR33* APA that generates them remain unclear. Interestingly, though, generation of both V2 and V3 mRNAs was found to involve multiple PASs. Here, we studied V2 and V3 mRNA PAS usage under various conditions and investigated the mechanisms responsible for *WDR33* APA. Our results provide novel insights into APA, especially that which occurs within coding sequences and/or introns, with the potential to generate distinct protein isoforms.

## Results

### CFIm25 regulates V2 and V3 expression

**Figure 1.**
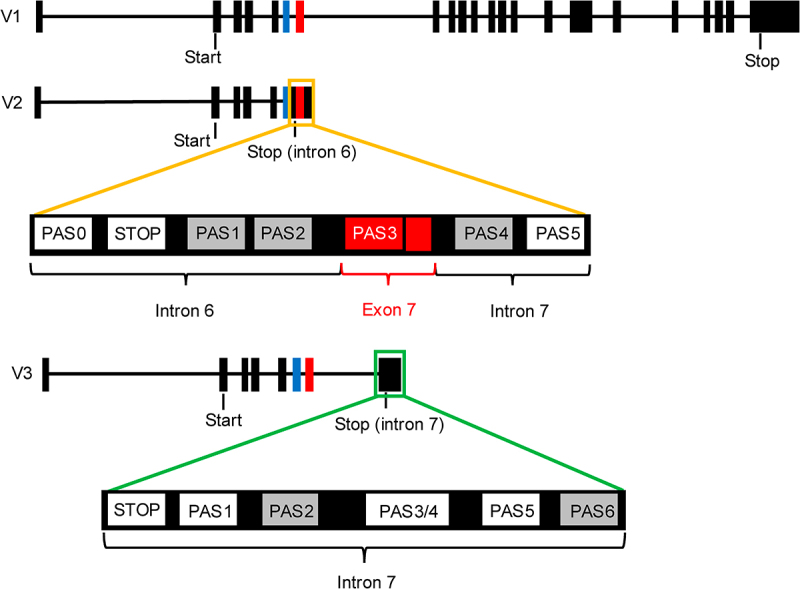
V2 and V3 are produced by APA.

In our previous study [16], we examined the genomic regions corresponding to V2 and V3 mRNA 3' ends and identified multiple A[A/U]UAAA hexamers. By 3' rapid amplification of cDNA end (3'RACE), three V2 hexamers and four V3 hexamers were found to be associated with a poly (A) tail, and therefore reflect functional PASs. The V2 PASs are located in introns 6 (PAS0) and 7 (PAS5), and in exon 7 (PAS3) (Figure 1), and usage of any of these PASs causes partial or full retention of intron 6, which contains an in-frame stop codon and encodes a C-terminal transmembrane domain (118 amino acids), producing the transmembrane V2 isoform [16]. The V3 active PASs are all clustered in intron 7 (PAS1, PAS3/4 and PAS5) (Figure 1). Usage of these PASs appears to be associated with the usage of an alternative 3' splice site (SS) within intron 7, leading to retention of a short sequence of intron 7, which also contains an in-frame stop codon [16]. The resultant V3 isoform only has a short V3-specific C-terminus (16 amino acids), which is not likely to contain a functional domain.

Schematic showing the exon-intron structures of V1 (top), V2 (middle) and V3 (bottom). V2 PASs are located within introns 6 and 7 and in exon 7 (shown in red). V3 PASs are clustered in intron 7. Not drawn to scale. Thick boxes: exons; horizontal lines: introns; blue box: exon 6; red box: exon 7; unshaded PASs: functional PASs; shaded PASs: non-functional PASs; Start: start codon; Stop: stop codon.

**Figure 2.**
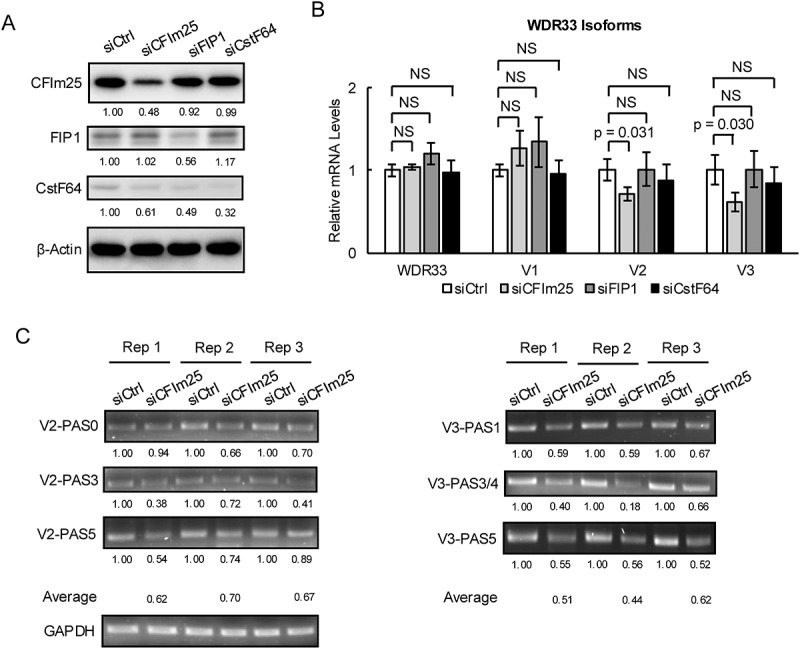
CFIm25 variably regulates V2 and V3 PAS usage.

To investigate mechanisms underlying V2 and V3 PAS usage, we first set out to identify possible *WDR33* APA regulator(s) by examining *WDR33* mRNA isoform expression upon siRNA knockdown (KD) in HeLa cells (KD efficiencies shown in Figure 2A) of three well-studied APA factors, CFIm25, FIP1 and CstF64 [12–14,19]. As detected by RT-qPCR, depletion of these three proteins did not significantly alter total *WDR33* or V1 mRNA expression (Figure 2B). However, KD of CFIm25, but not of FIP1 or CstF64, significantly downregulated total V2 (~30%) and V3 (~50%) mRNA expression (Figure 2B), indicating that CFIm25 is a regulator of *WDR33* APA.

A) Protein levels of CFIm25, FIP1 and CstF64 detected by western blots following knockdown by siRNAs in HeLa cells. The numbers indicate intensities of the bands in the membrane above normalized to the intensity of β-Actin. B) mRNA levels of total *WDR33*, V1, V2 and V3 in HeLa cells following CFIm25, FIP1 and CstF64 knockdown by siRNAs measured by RT-qPCR. Data is shown as means ± standard deviations (SD) from three replicates. NS: not significant. C) Usage of PASs of V2 (left) and V3 (right) in HeLa cells following CFIm25 knockdown detected by 3’RACE. The numbers indicate intensities of the bands in the gel above normalized to the intensity of GAPDH.

A UGUA motif, which is the binding site for CFIm25 [20], was identified in all V2 PASs (functional or non-functional). It is closer to the A[A/U]UAAA hexamer for both PAS0 and PAS3 (~20 nt upstream, appropriately positioned; see [21]) than for PAS5 and the non-functional PASs (>50 nt upstream). For V3, a UGUA motif was also identified in all functional and non-functional PASs. It is though located 6 nt downstream of A[A/U]UAAA for PAS1, which is not expected to enhance 3' end processing [21], and it is further upstream for all remaining PASs (>50 nt).

The above analysis suggests that CFIm25 levels might regulate different *WDR33* PASs differently. To test this, we performed 3'RACE to investigate changes of individual PAS usage in CFIm25-depleted cells. Notably, the effects of CFIm25 KD on V2 PASs were variable among three independent replicates (Rep) (Figure 2C). For PAS0, no change was observed for Rep1, but an ~30% decrease was observed for both Rep2 and Rep3. For PAS3, a ~ 60% reduction was observed for Rep1 and Rep3, while a ~ 30% decrease was detected for Rep2. For PAS5, a ~ 50% decrease was detected for Rep1, and decreases to a lesser extent were observed for both Rep2 (~25%) and Rep3 (~10%). Note, however, that the average reduction of these V2 PASs was ~30%, which is equivalent to the change of total V2 mRNA levels detected using RT-qPCR (although note that our 3'RACE analysis did not allow quantitation of the relative abundance of the different isoforms). Thus, despite consistently modulating V2 mRNA expression, CFIm25-mediated control of individual V2 PAS usage appears stochastic.

We next performed 3'RACE experiments with the V3 PASs (Figure 2C). CFIm25 KD consistently decreased PAS1 and PAS5 usage across all three replicates by ~40% and ~50%, respectively. For PAS3/4, a more drastic reduction for Rep2 (~80%) was detected than for Rep1 (~60%) and Rep3 (~40%). The average reduction among these V3 PASs was also consistent, at ~50%, which was, as with V2, similar to the reduction of total V3 mRNA levels detected by RT-qPCR. While it remains unclear how different UGUA motif positions affect *WDR33* PAS usage, our results together nevertheless suggest that CFIm25 KD affects V2 and V3 somewhat differently, and that there is no preferred PAS for either V2 or V3.

### V2 and V3 PAS usage is stochastic during immune responses

**Figure 3.**
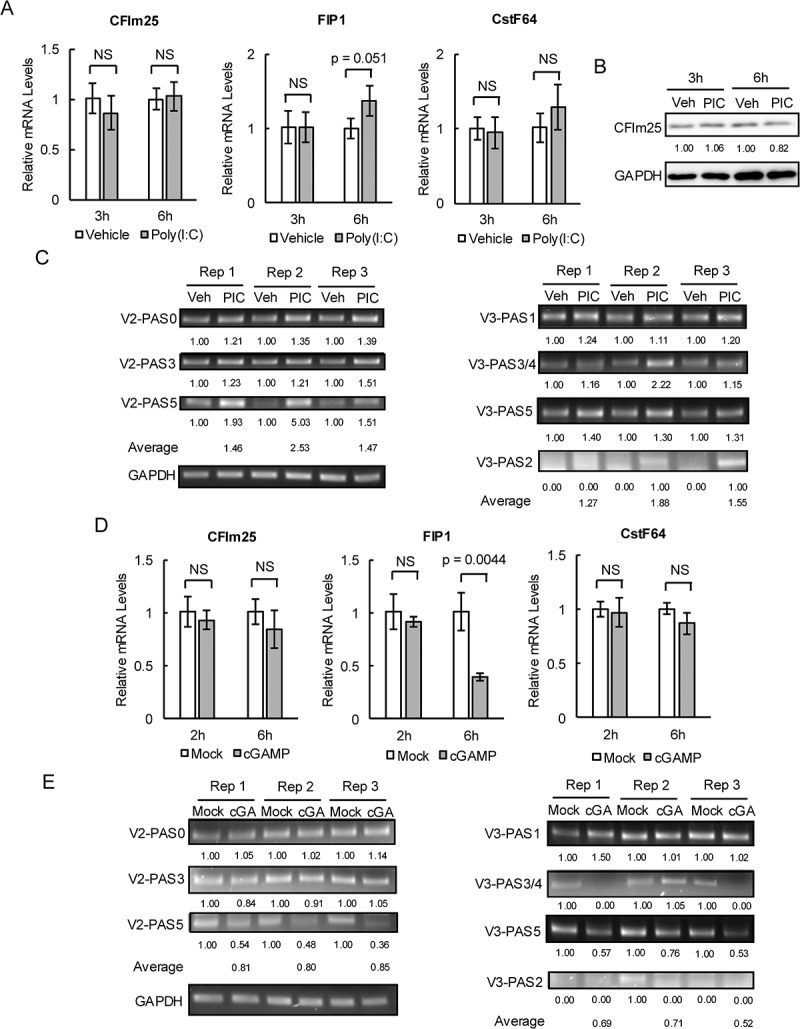
V2 and V3 PAS usage is stochastic under both dsRNA and dsDNA immune responses.

We previously reported that V2 and V3 mRNAs are both upregulated by ~2–3 fold during dsRNA immune responses but are both downregulated by ~50% following dsDNA stimulation [16]. We thus wished to determine how V2/V3 PAS usage changes under these conditions. First, we examined expression of CFIm25, FIP1 and CstF64 in HeLa cells following treatment with poly(I:C) to induce dsRNA-like immune responses. By RT-qPCR, we detected a ~1.5-fold increase (*p* = 0.051) in FIP1 mRNA levels 6 h after poly(I:C) treatment, but no significant changes for either CFIm25 and CstF64 at 3 and 6 h, or for FIP1 at 3 h (Figure 3A). No changes in protein levels for CFIm25 were observed by western blot at 3 and 6 h (Figure 3B). Thus, despite functioning to facilitate V2 and V3 mRNA expression in normally growing HeLa cells, CFIm25 is not responsible for poly(I:C)-induced V2 and V3 upregulation.

A) mRNA levels of CFIm25 (left), FIP1 (middle) and CstF64 (right) in HeLa cells treated with vehicle or poly(I:C) for 3 or 6 h measured by RT-qPCR. B) Protein levels detected by western blot of CFIm25 in HeLa cells treated with vehicle (veh) or poly(I:C) (PIC) for 3 or 6 h. The numbers indicate intensities of the bands in the membrane above normalized to the intensity of GAPDH. C) Usage of PASs of V2 (left) and V3 (right) in HeLa cells treated with vehicle (veh) or poly(I:C) (PIC) detected by 3’RACE. The numbers indicate intensities of the bands in the gel above normalized to the intensity of GAPDH. D) mRNA levels of CFIm25 (left), FIP1 (middle) and CstF64 (right) in cGAS^−/−^ BJ-5ta cells treated with mock or cGAMP for 2 or 6 h measured by RT-qPCR. E) Usage of PASs of V2 (left) and V3 (right) in cGAS^−/−^ BJ-5ta cells treated with mock or cGAMP (cGA) detected by 3’RACE. The numbers indicate intensities of the bands in the gel above normalized to the intensity of GAPDH. All bar graphs are shown as means ± SD from three replicates. NS: not significant.

Next, we performed 3'RACE to examine possible changes in V2 PAS usage following poly(I:C) stimulation in HeLa cells (Figure 3C). Usage of PAS0 and PAS3 was increased largely uniformly among three independent replicates, by ~20% to ~50%. While PAS5 usage was increased by ~50% for Rep3, ~2-fold and strikingly ~5-fold increases were detected for Rep1 and Rep2, respectively. PAS1, PAS2 and PAS4, which are non-functional under non-stimulated conditions [16], remained undetectable. Overall, usage of distinct V2 PASs following poly(I:C) stimulation was variable, but largely corresponded to the increase in total V2 mRNA (~2 fold), with PAS5 perhaps contributing to a greater extent to the total increase.

We also examined V3 PAS usage in poly(I:C)-stimulated HeLa cells (Figure 3C). PAS1 and PAS5 usage was increased relatively uniformly across replicates, by ~20% and ~30%, respectively. For PAS3/4, a ~ 2-fold increase was observed for Rep2, but the increases for Rep1 and Rep3 were only ~15%. Notably, usage of PAS2, which was non-functional under non-stimulated conditions [16], was observed in the poly(I:C)-treated cells. Overall, poly(I:C)-induced increases in V3 PAS usage were also variable, ranging from ~30% to ~90%. These increases were lower than the increase in V3 mRNA levels (~3-fold) [16], suggesting that additional, uncharacterized PASs may be used.

We next repeated the above experiments in cells under dsDNA immune responses. This was achieved by treating BJ-5ta immortalized human fibroblasts with 2’3’-cyclic GMP-AMP (cGAMP), which is an endogenous cellular second messenger produced by cyclic GMP-AMP synthase (cGAS) upon cytosolic dsDNA detection [22]. BJ-5ta was used because many commonly used cell lines do not respond to cGAMP [23]. In addition, the BJ-5ta cells we used were cGAS^−/−^ [24] and [16], such that the immune responses we induced were solely due to the added cGAMP. No changes in CFIm25 or CstF64 mRNA expression were detected at 2 and 6 h following cGAMP stimulation (Figure 3D). Thus, altered CFIm25 levels are also not responsible for cGAMP-induced V2 and V3 downregulation. While FIP1 expression was unchanged at 2 h, we observed an ~70% reduction in FIP1 at 6 h (Figure 3D). However, given that FIP1 levels do not affect V2 and V3 expression under non-stimulated conditions, FIP1 is likely not responsible for cGAMP-induced V2 and V3 downregulation.

We next examined V2/V3 PAS usage following cGAMP stimulation, again by 3’RACE (Figure 3E). No changes were detected for either V2 PAS0 or PAS3 across replicates. However, a consistent 40%–50% reduction was observed for V2 PAS5, indicating that PAS5 was the main V2 PAS affected by a dsDNA immune response. For V3, a relatively uniform decrease, 30%–40%, was detected for PAS5. PAS1 usage was unchanged for Rep2 and Rep3 but was paradoxically increased by ~50% for Rep1, while PAS3/4 usage was essentially completely eliminated for Rep1 and Rep3, but remained unchanged for Rep2. Surprisingly, PAS2, which was not found to be active by 3’RACE under non-stimulated conditions in HeLa cells [16], was detected in BJ-5ta cells without cGAMP in Rep2, and its usage was also completely eliminated following cGAMP stimulation. V3 PAS usage is thus also highly stochastic upon cGAMP treatment.

### V3 PASs are stronger than V2 PASs

**Figure 4.**
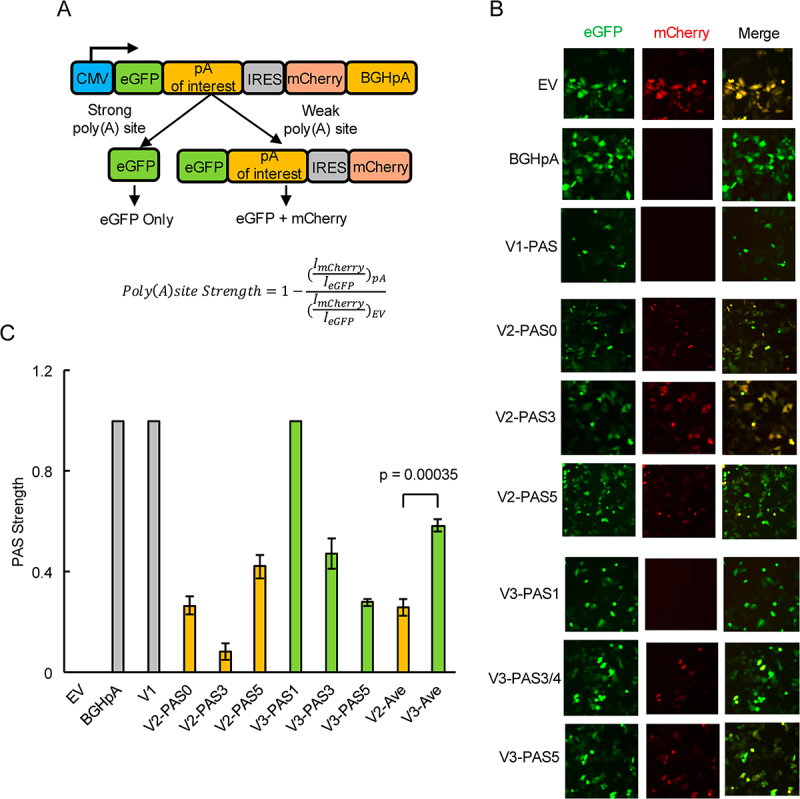
V3 PASs are stronger than V2 PASs.

Our findings above suggest that, in general, there is no preferred V2 and V3 PAS, under any of the conditions we examined. We were thus interested in determining the relative strength of these PASs, and in particular what drives usage of V2 vs V3 PASs. This was investigated using a modified PAS strength reporter plasmid, based on one described previously [25]. The original plasmid contains the open-reading frames (ORFs) of luciferases from two different species, with an internal ribosome entry site (IRES) inserted between them. A PAS of interest is then cloned between the upstream luciferase ORF and the IRES. In our modified reporter, we replaced the luciferases with eGFP and mCherry for easier detection by fluorescence microscopy. We inserted various PASs between the eGFP ORF and the IRES, such that eGFP is always expressed, but expression of mCherry, which is downstream of the IRES, is inversely proportional to the strength of the PAS (Figure 4A). PAS strength was quantified on a scale from 0 (weakest, no PAS control) to 1 (strongest, no detectable mCherry signal) (see Figure 4A, bottom panel). HeLa cells transfected with the empty vector (EV, containing no PAS insert) exhibited strong mCherry signals (PAS strength of 0), but no mCherry signals were detected for cells transfected with a derivative containing the strong bovine growth hormone PAS (BGHpA) (PAS strength of 1) [26], indicating that our modified reporter functioned as expected (Figure 4B,C).

A) Schematic of PAS strength reporter plasmid (top) and the formula used to calculate PAS strength (bottom). I: fluorescent signal intensity; pA: poly(A) site. B) eGFP and mCherry signals in HeLa cells transfected with control reporter plasmids (EV, BGHpA and V1; top), V2 PAS reporter plasmids (middle) and V3 PAS reporter plasmids (bottom). C) Quantification of PAS strength shown in (B) determined by the formula from (A). Grey bars: control PASs (EV, BGHpA and V1); Orange bars: V2 PASs; Green bars: V3 PASs; V2-Ave: average strength of the V2 PASs; V3-Ave: average strength of the V3 PASs. Data is shown as means ± SD from three replicates. A total of 600–1000 cells were quantified in each replicate.

We next cloned V1, V2 and V3 PASs into this reporter, and transfected the resultant plasmids into HeLa cells to evaluate strengths of these PASs (Figure 4B,C). The V1 PAS was very strong, as no mCherry signals were observed. V2 PAS0 and PAS5 were of moderate strength, at ~0.26 and ~0.42, respectively. V2 PAS3, which is located in exon 7, was very weak at ~0.08. For V3, PAS3/4 and PAS5 were also of moderate strength, at ~0.47 and ~0.28, respectively. No mCherry signals were observed for V3 PAS1, indicating that this is a very strong PAS. Overall, these experiments demonstrate that V3 PASs (average strength ~ 0.58), especially PAS1, are in general stronger than V2 PASs (average strength ~ 0.26).

### V2 is produced using weak PASs because intron 6 is removed inefficiently

**Figure 5.**
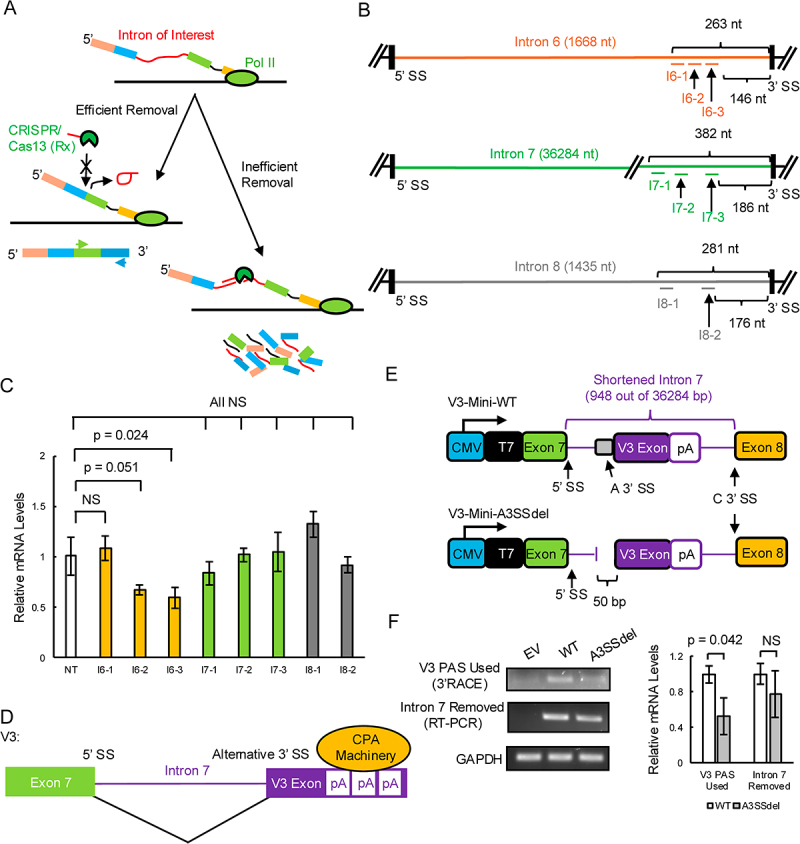
Splicing is involved in V2 and V3 mRNA production.

We previously reported that the abundance of V2 mRNAs is ~ 10-fold of that of V3 mRNAs [16], a result consistent with several other studies [27,28]. This appears to contradict the above findings that V3 PASs are stronger than V2 PASs. Given that V2 and V3 PASs are mainly located within introns, we hypothesized that V2 and V3 expression is also dependent on splicing efficiency. We thus used CRISPR/Cas13 RNA targeting to evaluate this possibility. Cas13 is an endonuclease that cuts RNAs complementary to its guide RNA (gRNA) [29]. We reasoned that if an intron of interest is removed inefficiently, it will remain in the pre-mRNA for a longer time than one that is spliced efficiently. Cas13 targeting such an intron would then have greater time to recognize and degrade the pre-mRNA, leading to a reduction in mRNA levels quantifiable by RT-qPCR (Figure 5A).

A) Schematic of intron removal efficiency quantification by CRISPR/Cas13 (Rx). B) Schematic showing the targeting locations of intron 6 gRNAs (I6-1, I6-2 and I6-3; top), intron 7 gRNAs (I7-1, I7-2 and I7-3; middle) and intron 8 gRNAs (I8-1 and I8-2; bottom). Not drawn to scale. C) *WDR33* mRNA levels detected by RT-qPCR in HeLa cells transfected with the indicated Cas13 gRNAs. White bar: non-targeting control gRNA (NT); Orange bar: intron 6 gRNAs; Green bars: intron 7 gRNAs; Grey bars: intron 8 gRNAs. Data is shown as means ± SD from three replicates. NS: not significant. D) Schematic showing that V3 mRNA production involved both alternative 3’SS usage and APA. Not drawn to scale. pA: polyadenylation site. E) Schematics of V3 minigene constructs. A 3’SS: alternative 3’ splice site; C 3’SS: canonical 3’ splice site. F) V3 PAS usage (left, top) detected by 3’RACE and intron 7 splicing (left, middle) by RT-PCR of the minigenes from (E) in HeLa cells. Quantifications (right) are shown as means ± SD from three replicates. NS: not significant.

We designed three non-overlapping gRNAs targeting intron 6, which is associated with V2, three non-overlapping gRNAs targeting intron 7, which is associated with V3, and two non-overlapping gRNAs targeting intron 8, which should not influence either V2 or V3 PAS usage. All gRNAs targeted regions ~150–400 nt upstream of the relevant 3' SSs (Figure 5B). We designed gRNAs to target these regions to avoid interfering with 3'SS recognition and to eliminate the potential effect of intron lengths. For instance, a long intron, such as intron 7 (36284 nt), would take longer time to be transcribed than a shorter intron, such as introns 6 and 8 (1668 nt and 1435 nt, respectively), before splicing can take place. As a consequence, a long intron would be more likely to be cleaved if the gRNAs target its 5' end or the middle region independent of splicing. *WDR33* mRNA levels were quantified using a primer pair detecting the exonic regions downstream (exons 16–18) of introns 6–8 by RT-qPCR. We reasoned that quantitation using primers upstream of introns 6–8 would be unreliable, since such primers also detect mature V2/V3 mRNAs, given that the gRNAs target regions downstream of V2/V3 functional PASs.

Using the above approach, we found that two of the three intron 6 gRNAs (I6–2 and I6–3) consistently reduced *WDR33* mRNA levels, by ~40% (Figure 5C). I6-1 gRNA, however, did not alter *WDR33* levels, perhaps because it was an inefficient gRNA. In contrast, no reductions in *WDR33* mRNA levels were detected for all gRNAs targeting introns 7 or 8 (Figure 5C). These results indicate that splicing of intron 6 is inefficient, as compared to intron 7 (and 8), which allows V2 mRNA to be produced using its weak PASs.

### Usage of strong V3 PAS is dependent on an intron 7 alternative 3’SS

V3 mRNAs are produced using an alternative 3’SS in intron 7 together with one of the active intronic PASs [16] see Figure 5D). It is unclear if these two events are linked. To address this, we generated two V3 minigene constructs (Figure 5E). The wildtype (WT) construct consisted of exons 7 and 8, and a shortened intron 7 (948 bp, full-length intron 7 is longer than 36 kb) containing the original 5' and 3'SSs, the alternative 3'SS, V3 exon and V3 PAS1. The alternative 3’SS (50 bp) was removed in the other construct, which we termed A3SSdel.

We next transfected these two constructs into HeLa cells and evaluated intron 7 splicing by RT-PCR and V3 PAS usage by 3'RACE, both using the T7 forward primer to specifically detect minigene mRNAs (Figure 5E). Importantly, no significant difference between these constructs was detected with respect to intron 7 removal, indicating that the 50 bp deletion did not affect intron 7 splicing, and that the alternative 3'SS is not required for intron 7 splicing as a whole (Figure 5F). However, the usage of V3 PAS was significantly reduced, by ~50%, in A3SSdel compared to WT (Figure 5F). Thus, although V3 PAS1 is a strong PAS *per se*, its usage as an intronic PAS is dependent to a significant degree on the intron 7 alternative 3'SS. This dependency on alternative splicing, together with efficient intron 7 removal described above, likely contribute to the overall low V3 expression levels. This splicing dependency also ensures production only of V3, and not a derivative resulting in retention of intron 7 sequences downstream of exon 7 (which has never been detected).

## Discussion

In this study, we investigated the regulation and mechanism of *WDR33* APA. While APA within 3'UTRs is well-characterized [2,11], intronic/exonic APA remains more poorly understood. For 3'UTR APA, strength of a PAS is position-dependent. In general, the distal (3'-most) PAS is the strongest, while the proximal (5’-most) PAS is weak, due for example to a weak G/U-rich element [15], suboptimal hexamer sequence [30], or the lack of a CFIm25-interacting UGUA motif [21]. Thus, a proposed unifying model for 3'UTR APA is that PAS choice is dependent on expression levels of CPA factors, such as CstF64, CFIm25 and FIP1 [13,15,21].

Our data indicates that the above model does not apply to *WDR33* intronic/exonic APA. Although we found that CFIm25 levels regulate expression of *WDR33* V2 and V3 isoforms, we also showed that regulation of *WDR33* internal PAS usage is stochastic. Notably, V2 and V3 PAS usage is also largely stochastic in dsRNA and dsDNA immune responses, and is not influenced by CFIm25 levels, confirming that this is a general property of *WDR33* APA. It should be noted that the averages of PAS usage fold changes were generally lower than the fold changes of total *WDR33* mRNA levels following both poly(I:C) and cGAMP stimulation. This was likely due to usage of unidentified cryptic PASs [31] and/or to differences in stability of the different PAS isoforms [32]. Nevertheless, given the stochastic PAS usage, and hence nonuniform changes of 3’UTR length, V2 and V3 expression during immune responses is likely regulated through direct PAS usage instead of post-transcriptional 3'UTR RNA interference. Further studies are warranted to identify V2 and V3 regulator(s) during immune responses, and to clarify whether stochastic usage is a general feature of selection of one of multiple PASs within introns/exons.

Depletion of CFIm25 is known to induce global 3'UTR shortening in many different cell types [12,33–36]. We thus expect CFIm25-mediated regulation of V2 and V3 to be cell-type non-specific. Notably, CFIm25 has recently been suggested to function as a non-canonical transcription factor in human embryonic stem cells [37]. This raises a possibility that CFIm25-mediated regulation of V2 and V3 might involve transcriptional control of the *WDR33* gene. This is unlikely, however, given that CFIm25 depletion did not alter total *WDR33* mRNA levels. Nevertheless, it would be of value to examine whether CFIm25 can regulate *WDR33* transcription, and to examine CFIm25’s regulation of V2 and V3 in other cell lines, in future studies.

Splicing has long been proposed to be involved in intronic APA regulation [38,39]. Using the RNA-cutting CRISPR/Cas13 to target introns as part of a novel splicing efficiency assay, we found that *WDR33* intron 6, but not introns 7 or 8, is removed inefficiently, which contributes to the production of V2 using weak PASs. This finding not only confirms the role of splicing in intronic APA, but also establishes CRISPR/Cas13 as a useful tool for intronic APA studies. It is also known that intronic APA can involve alternative 3’SS usage [40,41]. For example, similar to V3, usage of an alternative upstream 3’SS within intron 5 of the human MDX3 pre-mRNA causes retention of a small portion of intron 5 that contains an in-frame stop codon and a PAS, and the resultant isoform was found to be overexpressed in glioblastoma [42]. Using the minigene assays we developed, we found that alternative 3’SS and PAS usage are linked for the production of V3, thus providing mechanistic insights into this type of mRNA processing.

**Figure 6.**
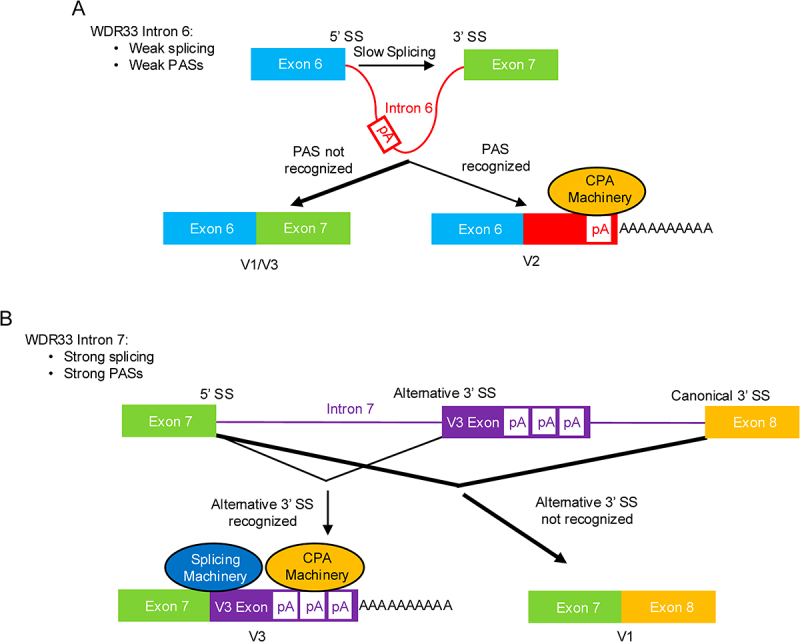
Mechanistic models of V2 and V3 mRNA production.

Together, our findings suggest a competition between the CPA and splicing machineries for V2 and V3 regulation, and based on this we propose the following mechanistic models. V2 is associated with intron 6, in which its C-terminal amino acids are encoded. Splicing of intron 6 is slow, and V2 PASs are weak (Figure 6A). Intron 6 tends to be removed before V2 PASs are recognized, resulting in pre-mRNAs destined for V1/V3. In a fraction of pre-mRNAs, V2 PASs are recognized before splicing, and V2 mRNA is produced (Figure 6A). Indeed, in all cases examined V2 mRNA is more abundant than V3 [27,28], and can be 50% or more as abundant as V1 [16]. V3 production involves both alternative 3’SS and PAS usage, which are coupled, within intron 7. Splicing of intron 7 is efficient, causing it to be removed in a majority of pre-mRNAs (Figure 6B). V3 mRNAs can only be produced when the alternative 3'SS is recognized, which in turn activates the downstream PASs (Figure 6B). These requirements for V3 likely contribute to its low expression levels. Thus, our models indicate that V2 and V3 can only be produced when PAS usage outcompetes splicing, and suggest that V2 and V3 could be regulated by the CPA and/or splicing machineries under different conditions. For instance, when CFIm25 is depleted, splicing of introns 6 and 7 outcompetes PAS usage, leading to the observed reductions in V2 and V3 levels. Overall, our models address not only how V2 and V3 are regulated but also the observed differences in their expression levels. We also note that the interplay between splicing and APA increases the opportunity for regulation of V2 and V3 expression, for which there is considerable evidence [16,27,28].

A) Schematic showing that splicing of intron 6 is slow, allowing V2 mRNAs to be produced using its weak poly(A) sites. pA: poly(A) site. B) Schematic showing that V3 mRNA production requires usage of both the alternative 3’ SS and poly(A) sites within intron 7, which together contributes to low V3 expression levels. pA: poly(A) site.

In summary, our study has revealed mechanisms underlying *WDR33* APA, an intriguing example of intronic/exonic APA that generates protein isoforms functionally unrelated to the primary protein product. We found that V2 and V3 mRNA PAS usage is highly stochastic under various conditions, and that *WDR33* APA is dependent on splicing efficiencies. It is also notable that while V2 and V3 are each created by APA, both can themselves undergo distinct patterns of APA. This provides novel avenues for regulation of the complex *WDR33* gene, and likely others with related structures. More broadly, our findings contribute to our general understanding of intronic/exonic APA, and illustrate that its underlying mechanisms, and regulation, can be distinct from those of 3'UTR APA.

## Materials and methods

### Cell culture and transfection

HeLa cells were from ATCC and were cultured in DMEM containing 10% FBS. PAS reporter plasmids, V3 minigenes (all generated in this study) and CRISPR/Cas13 (Rx) plasmids from [29] (pXR001, Addgene #109049; pXR003, Addgene #109053) were transfected into HeLa cells by lipofectamine 2000 (Invitrogen). For PAS reporters, eGFP and mCherry ORFs and IRES were cloned sequentially into pcDNA3. The poly(A) site inserts were the 206-nt genomic regions centring at the A[A/T]TAAA [43]. For V3 minigenes, the WT construct was generated by sequentially cloning *WDR33* exons 7 and 8, V3 exon and PAS1, and fragments of intron 7 amplified from 293T genomic DNA into pcDNA3. The A3SSdel construct was derived from the WT construct. CRISPR/Cas13 gRNAs were designed using cas13design.nygenome.org [44]. gRNA sequences (anti-sense) are: NT GGTAATGCCTGGCTTGTCGACGC; I6–1 ATTATTAGTGCTAATGCTATCCA; I6–2 AAAAGAAGACTTCAACAGAGCAG; I6–3 CGTCAGCTAACATAGGCATCTCA; I7–1 TCACTCACTTCAGTTGTCCCACT; I7–2 TGTAAAGATTTTGAAGTGTCTCA; I7–3 GTCAAGACACACAGGAGCCAGTC; I8–1 AAATACAAAAATTAGCCAGGCGT; and I8–2 CAGAAATAATATGCACTGCCCAG. All selected gRNAs have a guide score (ranges from 0 to 1) between 0.75 and 1 (higher the score, higher the predicted cleavage efficiency). siRNAs were transfected using lipofectamine RNAiMAX (Invitrogen). siRNA sequences (anti-sense) are: siCFIm25 AAGUAUAAUUGGUAAGAGG [36]; siFIP1 UAACUUCAAGUCCCAUUCG [36]; and siCstF64 GCUGCCCGGGACUAAAGCC. siCtrl (non-targeting siRNA) was from Sigma-Aldrich.

### qPCR and 3’RACE

Immune-stimulated HeLa and cGAS^−/−^ BJ-5ta cDNA samples were from [16]. qPCR was performed using the StepOnePlus qPCR system with SYBR Green PCR Master Mix (Applied Biosystems). Primer sequences for CFIm25, FIP1 and CstF64 are: CFIm25-RT-F TGAAGTTGAAGGACTAAAACGCT; CFIm25-RT-R ACCAGTTACCAATGCAATCGTC; FIP1-RT-F TGGCGTAAACCTGGTGCTG; FIP1-RT-R TCAAGTCCCATTCGTATCCTCT; CstF64-RT-F CCAAAGGGTTATGGCTTCTGT; and CstF64-RT-R TTGTCCACTCGAAGTGCTCTC. 3'RACE procedures are described in Scotto-Lavino et al. [45] and Liu and Manley [16]. For CRISPR/Cas13 assays, sequences of the primer pair are: forward CCCCTTCAACCAGGAAGGAC and reverse GGCCCATGTGATGGTTTGGA. The reverse primer to detect V3 minigene intron 7 splicing anneals to exon 8, and the sequence is AAGACTCTGCCCAGTCTTGG. 3’RACE, T7 and GAPDH primer sequences are described in Liu and Manley [16].

### Western blot

Proteins dissolved in SDS sample buffer were separated in 10% SDS-PAGE and transferred to nitrocellulose membranes. Primary antibodies were: rabbit-anti-NUDT21 (CFIm25) (ABclonal, A4482); rabbit-anti-FIP1L1 (FIP1) (ABclonal, A7138), rabbit-anti-CstF64 [14]; rabbit anti-Actin (Sigma-Aldrich, A2066) and rabbit anti-GAPDH (Sigma-Aldrich, G9545). Following overnight 4°C incubations with primary antibodies, the membranes were washed three times with 0.1% tween in PBS. The membranes were subsequently incubated with secondary antibodies (Sigma-Aldrich) for 1 h at room temperature. The ChemiDoc MP Imaging System (Bio-Rad) was used to detect chemiluminescence signals from ECL (Kindle Biosciences).

### Fluorescence microscopy

Twenty-four hours following transfection with the PAS reporter plasmids, HeLa cells cultured on 12-well plates were imaged directly using the Nikon Eclipse Ts2-R FL Inverted Fluorescence Microscope (Nikon) under 5X objective. Three random fields, each containing ~200–300 cells, were imaged for each sample per experiment. A total of three independent experiments were performed. Exposure times were kept constant for all three experiments.

### Quantifications and statistical analyses

All quantifications were performed using FIJI ImageJ [46]. For PAS reporter assays, eGFP and mCherry signals from all imaged cells (~600–1000 per replicate) were quantified, and averages were taken for each sample per replicate. The formula used to quantify PAS strength is shown in Figure 4A, bottom panel. Student’s t-tests were performed in Microsoft Excel. A p-value less than 0.05 was considered significant.

## Data Availability

The data that support the findings of this study are available in Mendeley Data at http://doi.org/10.17632/ccbcxz87zp.1.
